# Optimal therapy of type 2 diabetes: a controversial challenge

**DOI:** 10.18632/aging.100646

**Published:** 2014-03-26

**Authors:** Angela Dardano, Giuseppe Penno, Stefano Del Prato, Roberto Miccoli

**Affiliations:** Department of Clinical & Experimental Medicine, Section of Diabetes and Metabolic Diseases, University of Pisa, Via Paradisa 2, 56124, Pisa, Italy

**Keywords:** type 2 diabetes mellitus, elderly, aging, polypharmacy, glycemic control, treatment

## Abstract

Type 2 diabetes mellitus (T2DM) is one of the most common chronic disorders in older adults and the number of elderly diabetic subjects is growing worldwide. Nonetheless, the diagnosis of T2DM in elderly population is often missed or delayed until an acute metabolic emergency occurs. Accumulating evidence suggests that both aging and environmental factors contribute to the high prevalence of diabetes in the elderly. Clinical management of T2DM in elderly subjects presents unique challenges because of the multifaceted geriatric scenario. Diabetes significantly lowers the chances of “successful” aging, notably it increases functional limitations and impairs quality of life. In this regard, older diabetic patients have a high burden of comorbidities, diabetes-related complications, physical disability, cognitive impairment and malnutrition, and they are more susceptible to the complications of dysglycemia and polypharmacy. Several national and international organizations have delivered guidelines to implement optimal therapy in older diabetic patients based on individualized treatment goals. This means appreciation of the heterogeneity of the disease as generated by life expectancy, functional reserve, social support, as well as personal preference. This paper will review current treatments for achieving glycemic targets in elderly diabetic patients, and discuss the potential role of emerging treatments in this patient population.

## INTRODUCTION

The global prevalence of type 2 diabetes mellitus (T2DM) is rapidly growing as a consequence of life-style changes, urbanization and population aging [1]. The increase of life expectancy in parallel with increasing risk of developing T2DM with advancing age is a significant driver of the diabetes epidemic [2-4]. Although elderly diabetic patients are widely represented in the clinical practice, focus on diabetes care in this age group is still relatively scarce, while the momentum for more clinical trials including older patients should be encouraged. In this regard, a recent analysis showed that only 0.6% of interventional trials in diabetes specifically targeted elderly patients, 30.8% excluded patients older than 65 years and the majority excluded those aged >75 years [5]. Therefore, an important limiting factor for producing specific evidence-based clinical guidelines for older people with diabetes is the need to extrapolate evidence from data obtained from younger adults. Moreover, the general viewpoint is that people aged 60+ years are part of the older population and the terms ‘elderly’ and ‘older people’ are often interchangeable; however, this definition can be quite arbitrary and misleading. While an age thresholds could be used to identify geriatric age, the connotation of the geriatric patient comes from a more “comprehensive” definition that must include the functional status, the number and severity of comorbidities, societal and economic parameters, and overall degree of frailty [6]. Therefore, the optimal management of diabetes in the elderly population must recognize the vast heterogeneity to which disease duration, diabetes complications, functional status, comorbid illnesses, and patient's setting contribute to modulate the degree of vulnerability [7]. Elderly diabetic patients frequently have functional disabilities, cognitive decline, increased rates of bone fracture, polypharmacy, and hypoglycemic events due to comorbid illnesses. It is known that diabetic patientswith macrovascular complications are more susceptible to develop frailty, which, in turn, is associated with increased mortality [8]. Moreover, elderly patients may move in and out states of illness and functional impairment on a regular basis [9]. All these factors contribute to make the optimal therapy of T2DM in geriatric patients a controversial challenge [10] and advocate for a personalized approach [4,11]. This view is in line with the modern overall approach to T2DM management [12,13]. Position statement of the American Diabetes Association (ADA) and the European Association for the Study of Diabetes (EASD) has recently suggested an individualized and tailored care for T2DM, taking into account several elements, including age, patient attitude and expected treatment efforts, risks potentially associated with hypoglycemia or other adverse events, disease duration, life expectancy, comorbidities and established vascular complications as well as resources and support system [14].

Moreover, the most recent guidelines, while emphasizing individualization of HbA1c targets, underline that age *per se* should not be an excuse for suboptimal metabolic control [4,14,15]. Indeed, although attention has rightly been paid to the risks of over treatment of hyperglycemia in older subjects exposing them to the risk of hypoglycemia, treatment burden, increased risk of mortality, the potential negative impact of untreated or undertreated hyperglycemia, must be recognized even in patients with short life expectancy as a cause for dehydration, electrolyte abnormalities, urinary incontinence, dizziness, falls and overall poor outcome [4].

Reaching the best risk-to-benefit ratio of anti-diabetic treatment in the elderly T2DM patients is, however, not a simple task as the heterogeneity of this population has not been yet fully addressed by proper clinical trials. Therefore, in this review, we will discuss pros and cons as well as limitation of information with respect to the elderly population of the available pharmacologic treatments.

## METHODS

The authors collected materials for this review from a search of PubMed using as filters keywords relating to T2DM management in older people. In addition, a manual review of the references lists from retrieved articles was also performed to find further articles. Papers were reviewed for relevance by abstract, selecting only English language articles. The final list of cited references was chosen on the basis of relevance to the topic of review.

### Epidemiology of diabetes in the elderly

Ageing population is a growing problem and an important risk factor for several chronic diseases such as diabetes mellitus (DM) [16,17]. The prevalence of diabetes among US adults aged ≥65 years ranges from 22% to 33%, depending on the diagnostic criteria used [18,19]. Current estimates indicate that in the US, 26.9% of people ≥65 years of age are diagnosed with diabetes [20]. The high prevalence of T2DM among the elderly has been confirmed in a prospective population-based study in The Netherlands, showing that elderly patients, aged 70 years and over, account for 50% of the type 2 diabetic population, supporting health-care planning for older people [21].

As the population ages and both overweight and obesity continue to rise, the prevalence of diabetes in the elderly is expected to further increase [18,22,23] amplifying the already high burden of disease and its related costs [24]. Already today, the prevalence of diabetes in nursing homes is particularly high and care for diabetes in this setting specific is often inappropriate or insufficient [25,26]. Moreover, diabetes in the elderly is a well-recognized cause of accelerated frailty, disability, hospitalization, institutionalization, and death, thus absorbing a growing fraction of healthcare resources [14,27,28].

### Overview on pathogenesis of diabetes in the elderly

Aging is a process characterized by a multifaceted interaction of genetic, epigenetic, and environmental factors [29]. Genetic variants have been shown to impact on human longevity, showing a strict association with both unsuccessful aging and diabetes [29-31]. A strong genetic predisposition to T2DM in the elderly is apparent as well though only some candidate genes have been identified [32,33]. The pathogenesis of T2DM is characterized by two major mechanisms: impairedβ-cell function and insulin resistance [34]. The former is the main defect observed in lean older subjects, while obese older patients have relatively normal insulin secretion but marked resistance to insulin-mediated glucose disposal [35]. The Cardiovascular Health Study showed that the association between some risk factors and incident DM varied significantly depending on whether DM was preceded predominantly by insulin resistance, β-cell dysfunction, or both, thus suggesting putative subtypes of DM with biologic and clinical implications [36]. With aging, glucose-stimulated insulin response tends to decline, impaired insulin secretion pulsatility is lost, decreased sensitivity to incretins develops, andβ-cell mass is reduced [17]. Many events contribute to the age-related loss of β-cell mass and function, including the age-associated mitochondrial dysfunction, as well as increased oxidative stress and inflammation [36-42].

Aging results in a progressive loss of muscle mass and strength called “sarcopenia” that has a complex etiology involving neuronal, hormonal, immunological, nutritional and physical activity mechanisms [43,44]. Muscle mass loss in the elderly is associated with an increased fat mass infiltration that has been shown to be associated with worsened insulin sensitivity [45]. In this regard, in a recent cross-sectional study of 301 non diabetic subjects with a mean age of 65.9 years, a strong association between insulin resistance and relative muscle mass has been described [46]. Similarly, an association between sarcopenia and insulin resistance, diabetes, and metabolic syndrome has been reported in a large Korean population, particularly in elderly participants [47]. The link between sarcopenia and insulin resistance is a complex one, most likely mediated by several factors (i.e. mitochondrial dysfunction, reactive oxygen species, subtypes of adipocytes, fat-associated inflammation and adipocytokines) as recently reviewed [48]. Anyway, though sarcopenia may be not the primary cause of skeletal muscle insulin resistance in the elderly subjects, loss of lean muscle mass can be considered a worsening determinant.

Moreover, poor dietary habits and decreased physical activity all contribute to reduce insulin sensitivity in older population [49]. Glucose metabolism in older people can also be affected by co-existing illnesses and polypharmacy [33]. Finally, autoimmune phenomena may play a role in the pathogenesis of T2DM in a subset of older patients [50].

Although understanding of the pathogenesis of type 2 diabetes has advanced rapidly, the underlying molecular mechanisms remain partially unknown even because they are multiple, complex and linked each other. There is a huge progress in aging research on the role of the nutrient sensor mammalian target of rapamycin (mTOR) in aging and age-related diseases, including insulin resistance [51]. mTOR integrates multiple signals including growth factors, hormones, and cellular energy levels to regulate protein translation and cell metabloism, and survival, thus mediating the nutrient effects on insulin resistance. The attractive link between mTOR and insulin signaling cascades suggests that mTOR could become a therapeutic target in insulin-resistant status, even if its clinical application in metablic diseases is still limited [51].

**Figure 1:**
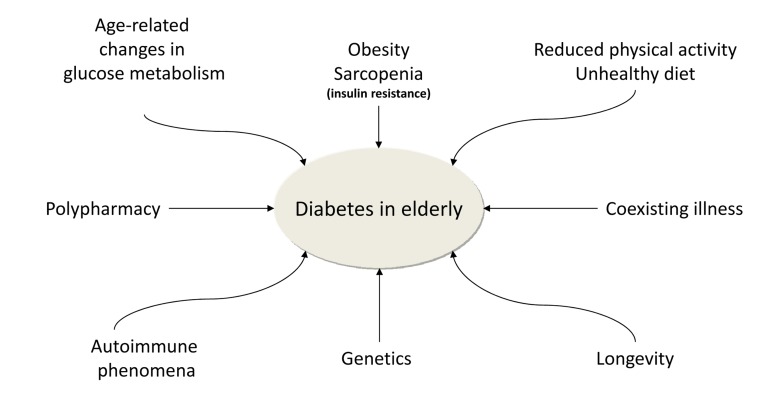
The figure 1 shows the main factors contributing to the high prevalence of diabetes mellitus in the elderly.

In summary, diabetes in the elderly is the result of a tangled and still incompletely understood combination of genetic and environmental factors that overlap and are magnified by the ageing process (Figure 1).

### Special thoughts for elderly patients affected by type 2 diabetes mellitus

T2DM can be independently associated with various aging phenotypes collectively defined as “geriatric syndromes” [11,52-57]. These geriatric conditions should be referred to as a *third category* of diabetic complications [58] and include cognitive impairment and dementia [55,59-67], depression [68,69], reduced muscle strength and quality [70-72], disability [73-77], falls and fall-related morbidity [78,79], as well as urinary incontinence [80]. These clinical conditions are very frequent in older diabetic people, especially in the frail ones. When present they exert a negative effect on the quality of life, functional outcomes, and mortality [54,81-83]. Moreover, these impairments, in particular cognitive decline, can affect in a significant manner the (self)-management of diabetes [84,85]. The cognitive decline is likely to initiate early in the natural progression of diabetes and it correlates with overall glycemic control [86-88]. To emphasize the link between T2DM and cognitive function some authors have proposed Alzheimer's disease as a third form of diabetes [89]. The etiology of cognitive impairment in diabetic patients is multifactorial [90] with a role played by dysglycemia, microvascular disease, insulin resistance, hyper-phosphorylation of tau protein, amyloid-β deposition, inflammation and oxidative stress [91,92]. More recently a role for sirtuins has been claimed. Sirtuins belong to a family of highly conserved protein deacetylases that depend on nicotinamide adenine dinucleotide (NAD+) for their activity [93]. These proteins have been shown to influence the course of several neurodegenerative disorders by controlling transcription factor activity [94,95]. Expression of SIRT1, the best characterized member within the family of sirtuins, seems to be reduced in T2DM and in conditions of insulin resistance [96], while, its activation improves insulin sensitivity [97].

Older diabetic people may be more vulnerable also because of coexisting comorbidities and related polypharmacy. Moreover, aging may be associated with changes in several pharmacokinetic and pharmaco-dynamic parameters. These include reduction of renal and hepatic function and increased volume of distribution of lipid soluble drugs resulting in an increase of drug half-life [98-101]. Pharmacodynamic changes can cause drug accumulation in the circulation and intensified sensitivity, for instance, to sulfonylureas thus increasing the risk of hypoglycemia [98]. In this setting, aging *per se* is a strong predictor of hypoglycemia [102-104] and hypoglycemia, in turn, is a major complicating factor of antidiabetic treatment [105]. Impaired counterregulatory response and increased symptom threshold worsen the risk and outcomes of hypoglycemia in elderly diabetic patients [106-107]. The risk of such an event in the elderly can be by reduced or irregular eating pattern, intercurrent diseases and concomitant use of other drugs [108]. Patients on five or more medications, particularly if they include ACE-inhibitors and nonselective beta-adrenoceptor antagonists, are more prone to drug-induced hypoglycemia [102-103]. Altogether, the various degree of concomitance of these factors may account for the variable rate of hypoglycaemia in the elderly reported in the literature. In the ACCORD trial, each one year increment in baseline age was associated with a 3% increase in the risk for hypoglycaemia requiring medical assistance [109].

Hypoglycaemia in the elderly is associated with serious morbidity, including cardiovascular events, stroke, arrhythmias, and falls result in fractures on a background of osteoporosis [110-117]. Results of post-hoc analyses of both the ACCORD and VADT trials have shown a strong association between severe hypoglycemia and cardiovascular mortality, especially in the elderly population [118]. In the ACCORD trial, intensive glucose lowering increased the risk of cardiovascular disease and total mortality in younger participants whereas it had a neutral effect in older participants, though the older subgroup had a greater annualized rate of severe hypoglycemic episodes [119]. Prevention of hypoglycemia requires identification of risk factors [102,113], patient and family education and reassurance regarding prevention, detection, and treatment of hypoglycemic events [4]. Reducing the risk of hypoglycemia should follow, in the elderly, the paradigm “start low and go slow” [100].

However, the heterogeneity of the older diabetic population must be fully appreciated [120] if adequate glycemic control has to be provided. Optimal care should balance health and function, tapering and tailoring the pharmacological approach in order to reach invidualized goals while avoiding clinical inertia. Biological rather than chronological age of the patient should be considered in defining therapeutic strategies [121]. Assessment of psychological age and social age is also recommended as part of a comprehensive (and multidisciplinary) geriatric appraisal of older people with diabetes in order to address the role of various comorbidities and polypharmacy before selecting treatment plans [122].

### Diabetes treatment guidelines for the elderly

Optimizing drug therapy is essential in the care of an older person. In line with the Quality Use of Medicines Framework, three key steps should be considered in drug prescription: 1) to identify the best treatment (considering non-pharmacological measures whenever possible); 2) to select medicines cleverly (considering the possibility of serious adverse effects or drug-to-drug interactions); and 3) to use medicines based on the strongest clinical evidences [123]. Likewise, three subgroups of older patients should be considered: 1) those who are relatively healthy; 2) those with complex medical histories in whom self-care may be difficult, and 3) those with a significant comorbidities and functional impairment [124,125]. Therefore, clinicians caring for older diabetic patients have to solve a therapeutic puzzle: balancing the patient's needs while taking into consideration health profile and glycemic goals and avoiding adverse effects.

Past guidelines have often not been able to provide specific recommendation for older diabetic patients. The California Healthcare Foundation/American Geriatrics Society in collaboration with other medical organizations suggested that a reasonable HbA1c goal for “relatively” healthy elderly with good functional status should be **≤**53 mmol/mol (<7%). On the contrary, for frail adults or with life expectancy <5 years, and when the risks of intensive glycemic control appear to overcome the benefits, a target HbA1c of 64 mmol/mol (8%) is suggested [54,126,127]. The U.S. Department of Veterans Affairs and the U.S. Department of Defense (VA/DOD) diabetes guidelines were updated few years ago. The VA/DOD guidelines do not distinguish by age-group, but stratify glycemic goals based on comorbidity and life expectancy [128]. The more recent ADA-EASD position statement suggests that the goals of treatment for older T2DM patients who are cognitively intact and have long life expectancy should be the same as those for younger subjects, while less stringent goals are suggested for those with limited life expectancy, advanced diabetes complications, or extensive comorbid conditions [14]. Recently, the International Association of Gerontology and Geriatrics, the European Diabetes Working Party for Older People, and the International Task Force of Experts in Diabetes recommended a target HbA1c range of 53-58 mmol/mol (7-7.5%) should be aimed for (DCCT aligned) for older T2DM patients with a single system involvement (Evidence level 1+, Grade of recommendation A), while for frailer (dependent; multisystem disease; care home residency including those with dementia) patients at high risk of hypoglycaemia in whom symptom control and avoidance of metabolic decompensation is paramount, target HbA1c range should range between 60-69 mmol/mol (7.6-8.5%) (Evidence level 1+, Grade of recommendation A) [15]. In the attempt to provide a less dictated approach a pragmatic strategy based on four variables has been recently proposed: (A)ge, (B)ody weight, (C)omplications and (D)uration of disease (ABCD) [121]. In short, in younger patients the goal is minimizing the risk of long-term complications while in older patients, especially in the frail ones the goal is to minimize shorter-term geriatric syndrome and maximize quality of life [127].

**Figure 2:**
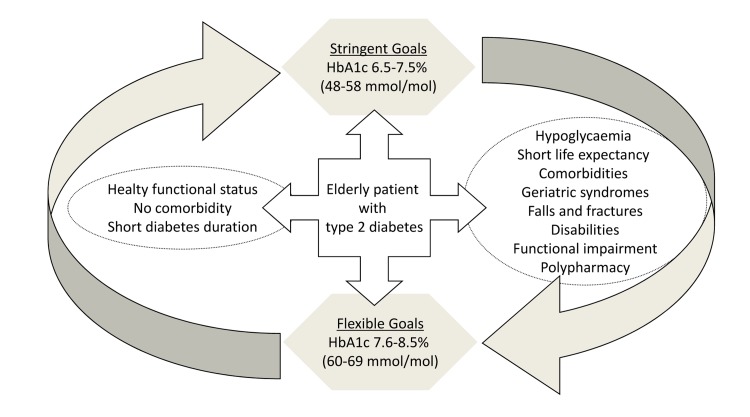
The figure 2 reports determinants of glycemic control in elderly patients affected by type 2 diabetes mellitus.

Determinants of glycemic control in elderly patients affected by T2DM are summarized in Figure 2.

### Treatment options

Mechanisms of action, advantages, disadvantages, concerns and novel aspects are summarized in the table.

**Table:** The table summarizes properties of currently available glucose lowering agents in elderly patients affected by type 2 diabetes mellitus.

**Sulfonylureas (1^st^ generation: glibenclamide 2^nd^ generation: glipizide, glimepiride, gliclazide**	Increased release of insulin by glucose independent closure of the ATP-sensitive K-channels	Proven glucose lowering efficacy Long term clinical experience Relatively low cost	Risk of hypoglycaemia Weight gain	Caution in renal impairment, hepatic dysfunction, concomitant insulin therapy, recent hospitalization, poor nutrition, cognitive decline and polypharmacy Cardiovascular profile is an important concern	
**Glinides (repaglinide)**	Increased release of insulin with a mechanism partially glucose dependent	Rapid onset of action and short duration Improved post-prandial hyperglycaemia	Risk of hypoglycaemia Weight gain Frequent dosing schedule Relatively high cost	Caution in hepatic dysfunction, concomitant insulin therapy, recent hospitalization, poor nutrition, cognitive decline and polypharmacy Cardiovascular profile is an important concern	
**Dipeptidyl peptidase-4 inhibitors (alogliptin, linagliptin, saxagliptin, sitagliptin, vildagliptin)**	Stimulation of insulin secretion Suppression of glucagon secretion	Low risk of hypoglycaemia	Increased respiratory infections Angio-edema Relatively high cost	Limited long term data Pancreatitis (?)	Potential cardio-protective and neuro-protective effects
**Glucagon-like peptide 1 analogues (exenatide, liraglutide, lixisenatide)**	Stimulation of insulin secretion Suppression of glucagon secretion Slow gastric emptying	Low risk of hypoglycaemia Weight loss	Gastro-intestinal effects (nausea/vomiting) Injectable Relatively high cost	Limited data on elderly patients Pancreatitis (?)	Potential cardio-protective and neuro-protective effects
**Sodium-glucose cotransporter 2 inhibitors (canaglifozin)**	Target Inhibition of renal reabsorption of glucose	Low risk of hypoglycaemia Systolic blood pressure reduction	Genitourinary infections, especially in women Pollakiuria Unintended weight loss	Cancer risk (?) Dose adjustment may be recommended in the elderly on loop diuretics and in those with an estimated GFR<60 mL/min or suffering from orthostatic hypotension	
**Insulin Fast-acting: insulin lispro Insulin aspart Insulin glulisine Long acting: Insulin glargine Insulin detemir Ultra long acting: insulin degludec**	Replacement of endogenous insulin	Mimics physiology Rapidly effective Theoretically unlimited efficacy	Risk of hypoglycaemia Injectable Weight gain	Require patient's ability or caregiver involvement Glucose monitoring and dose adjustment	

### Metformin

Metformin acts primarily through insulin sensitization of the liver, thus reducing hepatic glucose output and, secondarily, improving peripheral insulin resistance [129]. The recent ADA/EASD position statement and the AACE/ACE guidelines recommend metformin as first line treatment drug, due to its effectiveness and low risk of hypoglycemia [14,130]. In line with these recommendations, EDWPOP clinical guidelines stated that “age per se is not a contraindication to metformin” (Evidence level 2++; grade of recommendation B) [15] with some reduction in all-cause and cardiovascular mortality as compared to sulfonylurea monotherapy [131]. Metformin can cause gastrointestinal side effects (i.e. nausea, diarrhea, vomiting, abdominal pain), usually related to rapid titration and high dose initiation [132]. These adverse effects, although transient, may be undesirable in older, frail, anorexic and underweight patients [133], and may represent a dose-limiting barrier [134]. Metformin can also result in poor vitamin B12 status [135-137], which might accelerate cognitive dysfunction [138]. However, the main concern with respect to the use of metformin in the older adult with diabetes relates to the frequent existence of impaired renal function that should be regularly monitored in all patients on the drug [14,15,139]. EDWPOP guidelines recommend avoiding metformin “in patients with renal impairment and severe coronary, cerebrovascular or peripheral vascular disease” (Evidence level 2++; grade of recommendation B) [15] because of the risk of lactic acidosis. However, the observed association between metformin and lactic acidosis may be coincidental rather than causal [140], and there is no evidence from prospective comparative trials or from observational cohort studies that the risk of lactic acidosis and lactate levels differ appreciably in patients taking metformin as compared to other glucose-lowering treatments [141]. In a recent cross sectional analysis, it has been demonstrated that in elderly diabetic patients with moderate to severe renal impairment, the prescription of oral anti-hyperglycemic treatments is frequent, although inappropriate or not recommended. Nonetheless, metformin was associated with lower cardiovascular event rates [142]. The dose of metformin should be reduced (by 50% or to half-maximal dose) in patients with a GFR 30 to 45 mL/min per 1.73 m^2^, and monitored at 3-month interval and the drug stopped for an eGFR <30 ml/min per 1.73 m^2^ [143,144].

Metformin continues to gain attention for potential anti-cancer properties and neuro-protective effects, thus emerging as a novel therapeutic agent in aging-related diseases [132].

### Thiazolidinediones

Thiazolidinediones (TZDs) are peroxisome proliferation activated receptor-gamma (PPAR-gamma) agonists that improve peripheral insulin sensitivity by increasing peripheral adipose tissue lipogenesis and reducing hepatic fat content and hepatic glucose production [145]. TZDs could be an appealing strategy in the geriatric practice because of the low risk of hypoglycemia in comparison with sulfonylureas [146]. However, the real use of TZDs in the elderly is limited by their potential side effects. As suggested by two large observational studies, women taking TZDs have an increased risk of fractures as compared to those treated with other oral anti-diabetic drugs [147,148]. Similarly, TZDs have been associated with a questionable risk of bladder cancer [149-151]. Finally an increased prevalence of congestive hearth failure has been reported in patients on TZDs [152]. More debated remain the association between TZD use and cardiovascular events. In a case-control study of a cohort of 159,026 patients aged ≥ 66 years, TZD therapy (primarily rosiglitazone) was reported to be associated with increased risk of congestive hearth failure, acute myocardial infarction and death as compared to other oral hypoglycemic agents [153]. These results have not been confirmed in other studies [154] while an observational study suggested that TZDs were not associated with increased mortality for cardiac events and congestive heart failure in older patients [155]. The PROACTIVE prospective study showed a modest reduction of cardiovascular end points associated with pioglitazone [156] especially in patients with no peripheral artery disease [157].

In a 6-month randomized, open-controlled trial, it was reported that treatment with pioglitazone in diabetic patients affected by Alzheimer disease was associated with improvement in both cognition and regional cerebral blood flow, suggesting that PPAR-gamma agonists may offer a novel strategy for treating cognitive dysfunction [158].

Anyway, TZDs should not be considered a first-line approach and should be avoided in patients at high risk of weight gain, peripheral edema, heart failure, and bone loss and in those with history of osteoporosis or bladder cancer.

### Alpha-glucosidase inhibitors

Alpha-glucosidase inhibitors (AGIs) specifically target postprandial hyperglycemia by reducing carbohydrate digestion and absorption [159]. The hypoglycemic effect of AGIs is non as powerful, lowering HbA(1c) by 0.5-1%, but the risk of hypoglycemia, when they are used in monotherapy, is low, making these agents of interest in the elderly [35]. In older diabetic patients inadequately controlled on diet alone, acarbose has been associated with a significant reduction in HbA1c (−0.6%) as well as in the incremental post-prandial glucose values (−2.1 mmol/L) and mean fasting plasma glucose (−0.7 mmol/L) as compared to placebo [160]. In patients on prandial insulin, a mismatch between peak serum glucose level and peak prandial insulin levels may occur, with a possible increased risk of hypoglycemia [98], but a randomized, open-label study comparing the efficacy and safety of preprandial insulin in combination with acarbose in elderly patients (≥60 years) showed that addition of acarbose allows insulin adjustment from 30 min to immediately before meals, without affecting glycemic control, along with a low incidence of hypoglycemia [161]. However, the gastrointestinal adverse effects (i.e. abdominal bloating, flatulence, and diarrhea), frequent dosing, and relatively high costs [58] represent a limitation for the use of AGIs in geriatric patients

### Insulin secretagogues

Insulin secretagogue therapy (sulfonylureas, SUs and glitinides) is commonly used in clinical practice. These agents may be utilized as first, second-line or adjunct therapy behind metformin for treatment of T2DM. SUs cause glucose independent closure of the ATP-sensitive K-channels and release of insulin by binding to the SUR1 receptor on pancreatic beta cells [162,163]. Meglitinides, including repaglinide, have a similar mechanism but are partially glucose dependent and characterized by a rapid onset time and shorter duration of action [164,165]. EDWPOP guidelines for T2DM suggest adding an insulin secretagogue when glycemic targets have not been achieved or maintained (evidence level 1+; grade of recommendation B), avoiding glibenclamide in elderly persons (>70 years) with newly diagnosed T2DM because of marked risk of hypoglycaemia (evidence level 1+; grade of recommendation A) [15]. Renal impairment, hepatic dysfunction, concomitant insulin therapy, recent hospitalization, advanced age, poor nutrition, cognitive decline and polypharmacy are the main risk factors of hypoglycemia [102,166]. However, difference in such a risk exists among SUs with gliclazide being associated with lowest rate of hypoglycemia. Therefore, gliclazide, glipizide and third generation SUs (i.e. glimepiride) have been suggested as preferred agents in the elderly [102,166,167]. Accordingly, the American Geriatric Society clinical guidelines suggest short acting anti-diabetic agents to be preferred to longer-acting SUs [15,54].

A 24-week, randomized, open label, crossover trial, in elderly patients aged ≥65 years and in a subgroup of 37 patients aged≥75 years showed that treatment with repaglinide was associated with fewer hypoglycemic events as compared with those treated with glibenclamide [168]. Nonetheless, the risk of hypoglycemia associated with the use of meglitinides is not trivial [169].

Finally, cardiovascular profile of SUs is an important concern because of their possible effects on ischemic preconditioning [170], though the true relevance of this phenomenon has not yet been fully demonstrated and it is largely based in animal studies. Nonetheless, in a retrospective analysis, an increase in overall mortality risk has been reported for sulfonylureas as compared to metformin [171]. To summarize, SUs should not be seen as first-line treatment choice in the elderly. Were a sulfonylurea introduced, it should be prescribed with caution, usually starting at half the usual dose and to be titrated gradually while providing the person with diabetes and his/her relatives adequate education with respect to the risk of hypoglycemia.

### Incretins

Dipeptidyl peptidase-4 inhibitors (DPP4-I), usually referred as gliptins, are a relatively novel class of oral anti-hyperglycemic agents that stimulate insulin secretion and suppress glucagon secretion in a glucose-dependent manner [172,173]. Due to their efficacy, low risk of hypoglycemia and good tolerability, gliptin-based therapy plays a novel role in the management of diabetes and appears a fitting and intriguing choice for older adults [152,174]. According to the DWPOP guidelines, DPP4-I should be considered as an add-on therapy to metformin when the use of a SU poses an unacceptable risk of hypoglycemia (evidence level 1+; grade of recommendation A) [15]. The potential benefits of DPP-4 in the elderly have been discussed in a number of reviews [2,58,169,175-177]. However, *ad hoc* studies to establish clinical efficacy of DPP4-I in elderly T2DM patients have been only recently performed. These trials confirm in these individuals as well significant reduction of HbA1c with no hypoglycemia, body weight gain or other side effects [178-186]. Trials have been also performed in patients with moderate and severe renal impairment. With appropriate dosage adjustment with the exception of linagliptin, which has no renal excretion, these studies too have confirmed a good benefit-to-risk ratio with no further loss in renal function nor increased rate of hypoglycemia [187-192]. In the recent INTERVAL study, a multinational, double-blind, 24 week trial, drug naïve or inadequately controlled diabetic patients aged ≥70 years were randomly assigned to vildagliptin (patient mean age 75.1 yrs) or placebo (patient mean age 74.4 yrs)while setting individualized treatment targets on the basis of age, baseline HbA1c, comorbidities and frailty status [193]. Vildagliptin-treated patients achieved targets in 52.6% of the cases versus 27% of placebo-treated group, with a significant, relevant reduction of HbA1c values and no emergence of new safety signals [193]. Vildagliptin is metabolized mainly in the liver so liver function monitoring should be recommended, even if a recent meta-analysis indicated that vildagliptin is not associated with increased risk of hepatic events [194]. In diabetic patients aged up to 80 years, linagliptin had significant glucose lowering effects, showing a well-tolerated profile, which may result particularly useful in the elderly [195-199]. In a recent randomized, double blind, parallel-group, multinational phase 3 study, including patients aged 70 years or older (mean age 74·9 yrs), linagliptin was effective in lowering glucose, with a safety profile similar to placebo [186]. Data on the safety and efficacy of saxagliptin in the elderly are limited. Karyekar *et al* performed a post hoc analysis of pooled data from five 24-week phase 3 trials including older patients aged ≥65 years [185]. Results showed that saxagliptin was effective and well tolerated either when used as monotherapy, as add-on therapy, or initial combination therapy with other anti-diabetes drugs. Hypoglycemia, not requiring medical intervention, was reported in 3.0% to 9.4% of patients taking saxaglipitn (0%-8.0% for comparators) while confirmed hypoglycemia occurred in 0% to 0.7% (0% to 0.7% for comparators) [185]. Similar results have been reported for aloglipitn (as monotherapy and co-administration with other anti-diabetic agents) in a pooled analysis of six randomized, double blind, placebo-controlled studies, comparing the efficacy and safety of alogliptin in elderly (mean age 70 yrs) and younger (mean age 51.8 yrs) patients with type 2 diabetes mellitus [179]. The efficacy and tolerability of sitagliptin have been evaluated in a randomized, double blind, placebo-controlled, parallel-group study in diabetic patients aged ≥65 years (mean age 72 yrs). Although a relatively small number of subjects has been included, the study demonstrated that sitagliptin significantly improved glycemic parameters, with no hypoglycemia [184].

Over the last years more information on the potential of this class of oral agents have been gathered to further enhance the interest with respect to their use in elderly patients. Recent data suggest that gliptins may have pleiotropic, additional non-glycemic properties, including cardio- [200] and neuro-protective effects [201-204]. Cardiovascular (CV) safety of gliptins is a field of growing interest. In the SAVOR-TIMI trial while no effect on overall CV morbidity and mortality was reported, an excess of hospitalization for heart failure was observed [205]. This finding will require further assessment as absolute number of events was limited and an imbalance in the pro-BNP levels was present in the sitagliptin vs the control group with increased hospitalization occurred in the patients in the top pro-BNP quartile. Results from EXAMINE showed that among diabetic patients who had had a recent acute coronary syndrome, the rates of major adverse cardiovascular events were not different between patients on alogliptin as compared with those on placebo [206]. Taken together, these data confirm a neutral cardiovascular safety profile in high CV risk patients, as often are older T2DM individuals.

Glucagon-like peptide 1 (GLP-1) analogues include exenatide, liraglutide, and lixisenatide. These compounds may have a slightly greater effect on HbA1c as compared to DPP4-I, low risk of hypoglycemia and cause a moderate loss in body weight [207]. According to the DWPOP guidelines GLP-1 agonist may be considered as 3^th^ line add-on therapy to metformin and SU (evidence level 2++, grade of recommendation B) in very obese older patients up to an age of 75 years) [15]. Data on these agents above this age, especially the *frail patient*, are scanty [98,208]. In a placebo-controlled, patient-blind, crossover study compared elderly patients (mean age 78 ± 3 yrs) to controls (mean age 57 ± 6 yrs) with T2DM, exenatide was tolerated in all age groups, and dosage adjustment was requested according to patient's renal function [209]. A pooled analysis of 6 randomized, placebo-controlled, multinational trials included data from 3967 patients aged 18 to 80 years has shown that liraglutide provides effective glycemic control and is well tolerated in patients aged ≥65 years [210], although its use can be limited by concomitant impairment of the kidney function. Lixisenatide is the most recently approved GLP-1 receptor agonist [211,212], but less information, particularly in older T2DM patients, are available. Though preliminary data may suggest a potential neuro-protective effect [213,214], results of intervention trials are awaited. Importantly, clinical trials of the effects of GLP-1 agonists in patients with neurodegenerative diseases have been started, and clinical trials in patients with mild cognitive impairment or early-phase Alzheimer disease are on their way.

Although GLP-1 analogs are characterized by a favorable benefit-to-risk ratio, their use in the elderly should consider potential impairment of kidney function as well as gastric side effects. These compounds, though with some difference within the class, can cause nausea and vomiting and, therefore, may not be indicated in those elderly patients with erratic nutrition habits nor they may be recommended in those with lower body weight or progressive loss of body weight.

### Sodium-glucose co-transporter 2 inhibitors

Sodium-glucose cotransporter 2 (SGLT2) inhibitors represent a novel approach to treat T2DM. The mechanism of action is unique and does not hinge upon beta-cell function or tissue insulin sensitivity [215,216]. Through SGLT2 inhibition, reabsorption of tubular glucose is reduced and urinary glucose excretion increased [217] with very low risk of hypoglycemia. Moreover, loss of glucose causes mild but persistent reduction in body weight and concomitant fluid loss can reduce blood pressure. In a randomized, double-blind, placebo controlled phase 3 study, the efficacy and safety of canagliflozin, at different dosage, have been evaluated in older diabetic patients, aged 55 to 80 years (mean, 63.6 years), which were inadequately controlled on their usual treatment regimen [218]. The study demonstrated good tolerability and significant amelioration of glycemic control, along with a reduction of body weight and systolic blood pressure [218]. The most common side effects of these drugs include genitourinary infections and pollakiuria while, effects potentially dangerous for the geriatric age, such as dehydration, is uncommon. To note that concomitant prostate hypertrophy has not been so far identified as a limitation for the use of these agents [219,220]. Dose adjustment may be recommended in the elderly, those on loop diuretics, and those with an estimated glomerular filtration rate (eGFR) < 60 ml/min/1.73 m(2) if there are concerns or symptoms of volume-related side effects [221]. The efficacy of SGTL-2 inhibitors is likely to be self-limited in the presence of reduced GFR as this will be consensually associated with reduced tubular load and therefore reduced glucose excretion. Nonetheless, in a randomized, double-blind, placebo-controlled, phase 3 trial, canagliflozin improved glycaemic control and was generally well tolerated in subjects with T2DM and stage 3 chronic kidney disease [222]. Though SGLT2 inhibitors appear to be an emerging, appealing treatment option in diabetes, the safety issue remains the most important parameter determining the future of these drugs and more studies are needed before specific suggestions for elderly patients can be made.

### Insulin

The natural history of T2DM is characterized by a progressive decline in β-cell mass and function [223]. As a consequence, insulin therapy may be needed in older diabetic patients [98], also own to the anabolic effect the hormone. However, use of insulin requires special considerations in elderly patients. Comorbidities, cognitive dysfunction, vision loss, neuropathies, disabilities, and poor manual dexterity can all affect the patient's ability to self-management of insulin and may be limiting factors to insulin use in some cases or require caregiver assistance. Indeed, clinicians should evaluate the social network of patients who may have difficulty administering insulin - including family, friends, and caregivers - and should also determine whether the patient is socially isolated and/or living alone [224]. On the other hand, data show that insulin initiation in poorly controlled diabetic patients can improve quality of life [225]. Insulin initiation should be performed with caution and with defined targets to prevent worrisome adverse events, in particular hypoglycemia. As already stated, it is advisable to “start low and go slowly”, taking particular attention to insulin dose titration. Insulin requirements may be variable in elderly patients due to habits and pathophysiologic factors as well. While renal dysfunction may reduce renal insulin clearance and result in insulin accumulation with increased risk of hypoglycemia, concomitant use of other drugs can result in an increased insulin demand as it may occur with steroids. When oral agents fail to lower glucose levels adequately, insulin can be used either as monotherapy or in combination with a SUs or metformin (Evidence level 1+, grade of recommendation) [15]. Short acting insulin analogs (i.e insulin lispro, aspart, and glulisine) can help in the case of unpredictable eating habits or in those patients unable to adhere to dietary recommendations. Insulin dose can be titrated by carbohydrate intake also after meal when amount of carbohydrate ingested is erratic [224]. The long-acting basal insulin analogs (insulin detemir and glargine) represent by and large the most common choice for basal insulin therapy in elderly patients [14,15]. These analogs offer several advantages, including a more physiologic pharmacologic profile and they carry a reduced risk of hypoglycemia, particularly of nocturnal hypoglycaemia [226-228]. Focusing on elderly subjects, a pooled analysis of data from five randomized controlled trials showed that addition of insulin glargine to oral antidiabetic drugs in older adults (aged ≥65 years) with poor glycemic control was associated with greater reductions in HbA1c and fasting blood glucose and lower risk of nocturnal hypoglycemia as compared to NPH insulin [229]. Moreover, in a planned subgroup analysis of the original study, addition of once-daily morning glargine emerged as a simple regimen to initiate insulin therapy in elderly patients (aged 65 and older), restoring glycemic control more effectively and with less hypoglycemia than twice-daily 70/30 alone [230]. The new basal insulin analog, insulin degludec, has showed peculiar characteristics, including stable pharmacokinetic and pharmacodynamic profiles, true 24-hour duration of action in all patients, low within-person variability in absorption and glucose-lowering action, more flexible dose timing, and low occurrence of hypoglycemia [231]. In a recent pre-planned meta-analysis in elderly patients (≥ 65 years), the rate of confirmed and nocturnal hypoglycemia was significantly lower with insulin degludec as compared to insulin glargine [232]. Insulin premix formulations, in some elderly patients and in special settings, may provide added convenience [233]. Insulin pen devices may facilitate insulin injection and dosing and help patients maintaining their independence [224].

In summary, insulin remains the most effective and flexible form of treatment and as such it remains a valuable treatment opportunity in the elderly diabetic person, although insulin therapy in this group of subjects has not been adequately investigated. Moreover, the limited data comparing different insulin treatment schemes do not allow an evidence-based choice of insulin regimens in the elderly [234]. As repeatedly stated, careful individualization of insulin therapy as well is needed. While fast-acting insulin may be preferred to control post-prandial glucose, a carefully adjustment of dose on the basis of carbohydrate consumption is needed. Long-acting insulin can be easier to use though their long duration must be carefully taken into account as they may expose the elderly patient to the risk of late hypoglycemia. Careful education must be provided to the patient, his/her family and any person that may assist him/her. Finally, insulin may be necessary in concomitance with any intercurrent acute event, such as an infection, trauma, or surgical operation.

### Emerging drugs

Recent observations showing that a family of enzymes called sirtuins can significantly extend life in various organisms has led researchers to believe that they may also be capable to control age-related metabolic disorders, including obesity and type 2 diabetes in humans. It is well known that Sirtuin-1 (SIRT1) may have anti-diabetic effects, modulating insulin secretion and improving insulin resistance through several mechanisms [235]. Therefore, identification of therapeutic agents capable of modulating the expression and/or activity of sirtuins is expected to provide promising strategies for optimizing diabetes care [235]. In this regard, in vitro and animal studies have demonstrated that synthetic compounds, which have potent SIRT1-activating power, may improve insulin sensitivity in peripheral tissues (skeletal muscle and liver), reduce plasma glucose, and increase mitochondrial capacity [236,237]. Because of the sirtuin family's role in aging and age-associated pathologies, elderly subjects may represent the target populations for the treatment with this new class of compounds. Recently, elderly volunteers (mean age 67.1 yrs; range 61-77 yrs) were treated with SRT2104, a selective small molecule activator of SIRT1 or placebo once daily for 28 days, exploring multiple pharmacodynamic endpoints [238]. In this trial, although any significant changes in OGTT were observed, it has been demonstrated a trend toward a slower increase in insulin and C-peptide in the treated group [238]. The new molecule was generally safe and well tolerated and this observation supports further development of sirtuin activators to be used in future clinical trials in elderly patients.

## CONCLUSIONS

In spite of the fact that elderly diabetic patients account for a great majority of the diabetic population, limited attention has been paid in clinical trials thus limiting the clinical evidence on which treatment guidelines may find ground. Indeed, randomized trials generally have excluded older diabetic patients, in particular the *frail* ones, and clinical guidance has been largely based on data obtained in younger (adult) opulations, thus making the optimal therapy of T2DM in geriatric patients controversial. Older people with diabetes represent quite a heterogeneous population that is more likely to have comorbidities and geriatric syndromes, in addition to cardiovascular complications and hypo-glycemia. A more appropriate treatment of the elderly T2DM patients would require accurate definition of the geriatric patient not only based on clinical needs but also encompassing healthy, social, economic parameters. Clinicians must be fully aware of this heterogeneity in setting the therapeutic goals and in choosing the therapeutic strategy, which, invariably, should be centered on the patient's features, in line with the recent ADA/EASD position statement and in particular the ADA/AGS consensus document on diabetes in older adults [14,239]. These documents advocate a personalized and tailored care based on several elements, including patient's attitude and expected treatment efforts, risks potentially associated with hypoglycemia or other adverse events, disease duration, life expectancy, comorbidities and established vascular complications as well as resources and social and familial support. What must be always appreciate, however, is that age *per se* should not be an excuse for “clinical inertia” because the risk generated by inappropriate hyperglycemia has to be considered in all patients also in those with short life-expectancy. The complexity of interactions between comorbidity, polypharmacy, aging and settings where the older T2DM adult may be living must suggest caution as reflected by the classic adage “start low + go slow”. Yet, the targets and overall aim of the treatment should be always made clear.

## References

[R1] Chen L, Magliano DJ, Zimmet PZ (2011). The worldwide epidemiology of type 2 diabetes mellitus--present and future perspectives. Nat Rev Endocrinol.

[R2] Bourdel-Marchasson I, Schweizer A, Dejager S (2011). Incretin therapies in the management of elderly patients with type 2 diabetes mellitus. Hosp Pract (1995).

[R3] van Dieren S, Beulens JW, van der Schouw YT, Grobbee DE, Neal B (2010). The global burden of diabetes and its complications: an emerging pandemic. Eur J Cardiovasc Prev Rehabil.

[R4] Kirkman MS, Briscoe VJ, Clark N, Florez H, Haas LB, Halter JB, Huang ES, Korytkowski MT, Munshi MN, Odegard PS, Pratley RE, Swift CS (2012). Diabetes in older adults. Diabetes Care.

[R5] Lakey WC, Barnard K, Batch BC, Chiswell K, Tasneem A, Green JB (2013). Are current clinical trials in diabetes addressing important issues in diabetes care?. Diabetologia.

[R6] Global guideline for type 2 diabetes. IDF Clinical Guidelines Task Force.

[R7] Clegg A, Young J, Iliffe S, Rikkert MO, Rockwood K (2013). Frailty in elderly people. Lancet.

[R8] Espinoza SE, Jung I, Hazuda H (2012). Frailty transitions in the San Antonio Longitudinal Study of Aging. J Am Geriatr Soc.

[R9] Huang ES (2007). Appropriate application of evidence to the care of elderly patients with diabetes. Curr Diabetes Rev.

[R10] Chen LK, Chen YM, Lin MH, Peng LN, Hwang SJ (2010). Care of elderly patients with diabetes mellitus: a focus on frailty. Ageing Res Rev.

[R11] McLaren LA, Quinn TJ, McKay GA (2013). Diabetes control in older people. BMJ.

[R12] Del Prato S, LaSalle J, Matthaei S, Bailey CJ Global Partnership for Effective Diabetes Management. Tailoring treatment to the individual in type 2 diabetes practical guidance from the Global Partnership for Effective Diabetes Management. Int J Clin Pract.

[R13] Raz I, Riddle MC, Rosenstock J, Buse JB, Inzucchi SE, Home PD, Del Prato S, Ferrannini E, Chan JC, Leiter LA, Leroith D, Defronzo R, Cefalu WT (2013). Personalized management of hyperglycemia in type 2 diabetes: reflections from a Diabetes Care Editors' Expert Forum. Diabetes Care.

[R14] Inzucchi SE, Bergenstal RM, Buse JB, Diamant M, Ferrannini E, Nauck M, Peters AL, Tsapas A, Wender R, Matthews DR (2012). American Diabetes Association (ADA); European Association for the Study of Diabetes (EASD). Management of hyperglycemia in type 2 diabetes: a patient-centered approach: position statement of the American Diabetes Association (ADA) and the European Association for the Study of Diabetes (EASD). Diabetes Care.

[R15] Sinclair AJ, Paolisso G, Castro M, Bourdel-Marchasson I, Gadsby R, Rodriguez Mañas L (2011). European Diabetes Working Party for Older People. European Diabetes Working Party for Older People 2011 clinical guidelines for type 2 diabetes mellitus. Executive summary. Diabetes Metab.

[R16] Kaul K, Tarr JM, Ahmad SI, Kohner EM, Chibber R (2012). Introduction to diabetes mellitus. Adv Exp Med Biol.

[R17] Gong Z, Muzumdar RH (2012). Pancreatic function, type 2 diabetes, and metabolism in aging. Int J Endocrinol.

[R18] Narayan KM, Boyle JP, Geiss LS, Saaddine JB, Thompson TJ (2006). Impact of recent increase in incidence on future diabetes burden: U.S., 2005-2050. Diabetes Care.

[R19] Selvin E, Coresh J, Brancati FL (2006). The burden and treatment of diabetes in elderly individuals in the u.s. Diabetes Care.

[R20] Center for Disease Control and Prevention (2011). National diabetes fact sheet: national estimates and general information of diabetes and pre-diabetes in the United States. http//www.cdc.gov/diabetes/pubs/pdf/ndfs-2011.pdf.

[R21] Ubink-Veltmaat LJ, Bilo HJ, Groenier KH, Houweling ST, Rischen RO, Meyboom-de Jong B (2003). Prevalence, incidence and mortality of type 2 diabetes mellitus revisited: a prospective population-based study in The Netherlands (ZODIAC-1). Eur J Epidemiol.

[R22] Boyle JP, Thompson TJ, Gregg EW, Barker LE, Williamson DF (2010). Projection of the year 2050 burden of diabetes in the US adult population: dynamic modeling of incidence, mortality, and prediabetes prevalence. Popul Health Metr.

[R23] Caspersen CJ, Thomas GD, Boseman LA, Beckles GL, Albright AL (2012). Aging, diabetes, and the public health system in the United States. Am J Public Health.

[R24] Corriere M, Rooparinesingh N, Kalyani RR (2013). Epidemiology of diabetes and diabetes complications in the elderly: an emerging public health burden. Curr Diab Rep.

[R25] Bouillet B, Vaillant G, Petit JM, Duclos M, Poussier A, Brindisi MC, Vergès B (2010). Are elderly patients with diabetes being overtreated in French long-term-care homes?. Diabetes Metab.

[R26] Dybicz SB, Thompson S, Molotsky S, Stuart B (2011). Prevalence of diabetes and the burden of comorbid conditions among elderly nursing home residents. Am J Geriatr Pharmacother.

[R27] Morley JE (2008). Diabetes, sarcopenia, and frailty. Clin Geriatr Med.

[R28] Laiteerapong N, Karter AJ, Liu JY, Moffet HH, Sudore R, Schillinger D, John PM, Huang ES (2011). Correlates of quality of life in older adults with diabetes: the diabetes & aging study. Diabetes Care.

[R29] Vacante M, Malaguarnera M, Motta M (2011). Revision of the ADA-classification of diabetes mellitus type 2 (DMT2): the importance of maturity onset diabetes (MOD), and senile diabetes (DS). Arch Gerontol Geriatr.

[R30] Capri M, Salvioli S, Sevini F, Valensin S, Celani L, Monti D, Pawelec G, De Benedictis G, Gonos ES, Franceschi C (2006). The genetics of human longevity. Ann N Y Acad Sci.

[R31] Motta M, Malaguarnera M, Ferrari E, Mauro VN, Ferrucci L, Rapisarda R, Tomasello FB, Basile G, Ferlito L, Passamonte M, Bennati E (2007). Genealogy of centenarians and their relatives: a study of 12 families. Arch Gerontol Geriatr.

[R32] Canadian Diabetes Association. Clinical Practice Guidelines Expert Committee (2008). Can J Diabetes.

[R33] Meneilly GS, Tessier D (2001). Diabetes in elderly adults. J Gerontol A Biol Sci Med Sci.

[R34] Kadowaki T (2000). Insights into insulin resistance and type 2 diabetes from knockout mouse models. J Clin Invest.

[R35] Abdelhafiz AH, Sinclair AJ (2013). Management of type 2 diabetes in older people. Diabetes Ther.

[R36] Imamura F, Mukamal KJ, Meigs JB, Luchsinger JA, Ix JH, Siscovick DS, Mozaffarian D (2013). Risk factors for type 2 diabetes mellitus preceded by β-cell dysfunction, insulin resistance, or both in older adults: the Cardiovascular Health Study. Am J Epidemiol.

[R37] Ramsey KM, Mills KF, Satoh A, Imai S (2008). Age-associated loss of Sirt1-mediated enhancement of glucose-stimulated insulin secretion in beta cell-specific Sirt1-overexpressing (BESTO) mice. Aging Cell.

[R38] Chang AM, Halter JB (2003). Aging and insulin secretion. Am J Physiol Endocrinol Metab.

[R39] Cooksey RC, Jouihan HA, Ajioka RS, Hazel MW, Jones DL, Kushner JP, McClain DA (2004). Oxidative stress, beta-cell apoptosis, and decreased insulin secretory capacity in mouse models of hemochromatosis. Endocrinology.

[R40] Szoke E, Shrayyef MZ, Messing S, Woerle HJ, van Haeften TW, Meyer C, Mitrakou A, Pimenta W, Gerich JE (2008). Effect of aging on glucose homeostasis: accelerated deterioration of beta-cell function in individuals with impaired glucose tolerance. Diabetes Care.

[R41] Del Prato S (2009). Role of glucotoxicity and lipotoxicity in the pathophysiology of Type 2 diabetes mellitus and emerging treatment strategies. Diabet Med.

[R42] Licastro F, Candore G, Lio D, Porcellini E, Colonna-Romano G, Franceschi C, Caruso C (2005). Innate immunity and inflammation in ageing: a key for understanding age-related diseases. Immun Ageing.

[R43] Kim TN, Choi KM (2013). Sarcopenia: Definition, Epidemiology, and Pathophysiology. J Bone Metab.

[R44] Narici MV, Maffulli N (2010). Sarcopenia: characteristics, mechanisms and functional significance. Br Med Bull.

[R45] Goodpaster BH, Krishnaswami S, Resnick H (2003). Association between regional adipose tissue distribution and both type 2 diabetes and impaired glucose tolerance in elderly men and women. Diabetes Care.

[R46] Bijlsma AY, Meskers CG, van Heemst D, Westendorp RG, de Craen AJ, Maier AB (2013). Diagnostic criteria for sarcopenia relate differently to insulin resistance. Age (Dordr).

[R47] Moon SS (2014). Low skeletal muscle mass is associated with insulin resistance, diabetes, and metabolic syndrome in the Korean population: the Korea National Health and Nutrition Examination Survey (KNHANES) 2009-2010. Endocr J.

[R48] Krentz AJ1, Viljoen A, Sinclair A (2013). Insulin resistance: a risk marker for disease and disability in the older person. Diabet Med.

[R49] Scheen AJ (2005). Diabetes mellitus in the elderly: insulin resistance and/or impaired insulin secretion?. Diabetes Metab.

[R50] Pietropaolo M, Towns R, Eisenbarth GS (2012). Humoral autoimmunity in type 1 diabetes: prediction, significance, and detection of distinct disease subtypes. Cold Spring Harb Perspect Med.

[R51] Blagosklonny MV (2013). TOR-centric view on insulin resistance and diabetic complications: perspective for endocrinologists and gerontologists. Cell death & disease.

[R52] Lu FP, Lin KP, Kuo HK (2009). Diabetes and the risk of multi-system aging phenotypes: a systematic review and meta-analysis. PLoS One.

[R53] Vischer UM, Bauduceau B, Bourdel-Marchasson I, Blickle JF, Constans T, Fagot-Campagna A, Kaloustian E, Lassman-Vague V, Lecomte P, Simon D, Tessier D, Verny C, Doucet J (2009). Alfediam/SFGG French-speaking group for study of diabetes in the elderly. A call to incorporate the prevention and treatment of geriatric disorders in the management of diabetes in the elderly. Diabetes Metab.

[R54] Brown AF, Mangione CM, Saliba D, Sarkisian CA (2003). California Healthcare Foundation/American Geriatrics Society Panel on Improving Care for Elders with Diabetes. Guidelines for improving the care of the older person with diabetes mellitus. J Am Geriatr Soc.

[R55] Gregg EW, Engelgau MM, Narayan V (2002). Complications of diabetes in elderly people. BMJ.

[R56] Sinclair A, Morley JE, Rodriguez-Mañas L, Paolisso G, Bayer T, Zeyfang A, Bourdel-Marchasson I, Vischer U, Woo J, Chapman I, Dunning T, Meneilly G, Rodriguez-Saldana J (2012). Diabetes mellitus in older people: position statement on behalf of the International Association of Gerontology and Geriatrics (IAGG), the European Diabetes Working Party for Older People (EDWPOP), and the International Task Force of Experts in Diabetes. J Am Med Dir Assoc.

[R57] Volpato S, Maraldi C, Fellin R (2010). Type 2 diabetes and risk for functional decline and disability in older persons. Curr Diabetes Rev.

[R58] Kim KS, Kim SK, Sung KM, Cho YW, Park SW (2012). Management of type 2 diabetes mellitus in older adults. Diabetes Metab J.

[R59] Crooks VC, Buckwalter JG, Petitti DB (2003). Diabetes mellitus and cognitive performance in older women. Ann Epidemiol.

[R60] Grodstein F, Chen J, Wilson RS, Manson JE (2001). Nurses' Health Study. Type 2 diabetes and cognitive function in community-dwelling elderly women. Diabetes Care.

[R61] Gregg EW, Yaffe K, Cauley JA, Rolka DB, Blackwell TL, Narayan KM, Cummings SR (2000). Is diabetes associated with cognitive impairment and cognitive decline among older women? Study of Osteoporotic Fractures Research Group. Arch Intern Med.

[R62] Leibson CL, Rocca WA, Hanson VA, Cha R, Kokmen E, O'Brien PC, Palumbo PJ (1997). Risk of dementia among persons with diabetes mellitus: a population-based cohort study. Am J Epidemiol.

[R63] Luchsinger JA, Tang MX, Stern Y, Shea S, Mayeux R (2001). Diabetes mellitus and risk of Alzheimer's disease and dementia with stroke in a multiethnic cohort. Am J Epidemiol.

[R64] Velayudhan L, Poppe M, Archer N, Proitsi P, Brown RG, Lovestone S (2010). Risk of developing dementia in people with diabetes and mild cognitive impairment. Br J Psychiatry.

[R65] Cheng G, Huang C, Deng H, Wang H (2012). Diabetes as a risk factor for dementia and mild cognitive impairment: a meta-analysis of longitudinal studies. Intern Med J.

[R66] McCrimmon RJ, Ryan CM, Frier BM (2012). Diabetes and cognitive dysfunction. Lancet.

[R67] Fontbonne A, Berr C, Ducimetière P, Alpérovitch A (2001). Changes in cognitive abilities over a 4-year period are unfavorably affected in elderly diabetic subjects: results of the Epidemiology of Vascular Aging Study. Diabetes Care.

[R68] Ali S, Stone MA, Peters JL, Davies MJ, Khunti K (2006). The prevalence of co-morbid depression in adults with Type 2 diabetes: a systematic review and meta-analysis. Diabet Med.

[R69] Gavard JA, Lustman PJ, Clouse RE (1993). Prevalence of depression in adults with diabetes. An epidemiological evaluation. Diabetes Care.

[R70] Volpato S, Bianchi L, Lauretani F, Lauretani F, Bandinelli S, Guralnik JM, Zuliani G, Ferrucci L (2012). Role of muscle mass and muscle quality in the association between diabetes and gait speed. Diabetes Care.

[R71] Park SW, Goodpaster BH, Strotmeyer ES, Kuller LH, Broudeau R, Kammerer C, de Rekeneire N, Harris TB, Schwartz AV, Tylavsky FA, Cho YW, Newman AB (2007). Health, Aging, and Body Composition Study. Accelerated loss of skeletal muscle strength in older adults with type 2 diabetes: the health, aging, and body composition study. Diabetes Care.

[R72] Park SW, Goodpaster BH, Lee JS, Kuller LH, Boudreau R, de Rekeneire N, Harris TB, Kritchevsky S, Tylavsky FA, Nevitt M, Cho YW, Newman AB (2009). Health, Aging, and Body Composition Study. Excessive loss of skeletal muscle mass in older adults with type 2 diabetes. Diabetes Care.

[R73] Volpato S, Ferrucci L, Blaum C, Ostir G, Cappola A, Fried LP, Fellin R, Guralnik JM (2003). Progression of lower-extremity disability in older women with diabetes: the Women's Health and Aging Study. Diabetes Care.

[R74] Ryerson B, Tierney EF, Thompson TJ, Engelgau MM, Wang J, Gregg EW, Geiss LS (2003). Excess physical limitations among adults with diabetes in the U.S. population, 1997-1999. Diabetes Care.

[R75] Gregg EW, Beckles GL, Williamson DF, Leveille SG, Langlois JA, Engelgau MM, Narayan KM (2000). Diabetes and physical disability among older U.S. adults. Diabetes Care.

[R76] Wu JH, Haan MN, Liang J, Ghosh D, Gonzalez HM, Herman WH (2003). Diabetes as a predictor of change in functional status among older Mexican Americans: a population-based cohort study. Diabetes Care.

[R77] Kalyani RR, Saudek CD, Brancati FL, Selvin E (2010). Association of diabetes, comorbidities, and A1C with functional disability in older adults: results from the National Health and Nutrition Examination Survey (NHANES), 1999-2006. Diabetes Care.

[R78] Schwartz AV, Hillier TA, Sellmeyer DE, Resnick HE, Gregg E, Ensrud KE, Schreiner PJ, Margolis KL, Cauley JA, Nevitt MC, Black DM, Cummings SR (2002). Older women with diabetes have a higher risk of falls: a prospective study. Diabetes Care.

[R79] Strotmeyer ES, Cauley JA, Schwartz AV, Nevitt MC, Resnick HE, Bauer DC, Tylavsky FA, de Rekeneire N, Harris TB, Newman AB (2005). Nontraumatic fracture risk with diabetes mellitus and impaired fasting glucose in older white and black adults: the health, aging, and body composition study. Arch Intern Med.

[R80] Brown JS, Vittinghoff E, Lin F, Nyberg LM, Kusek JW, Kanaya AM (2006). Prevalence and risk factors for urinary incontinence in women with type 2 diabetes and impaired fasting glucose: findings from the National Health and Nutrition Examination Survey (NHANES) 2001-2002. Diabetes Care.

[R81] Inouye SK, Studenski S, Tinetti ME, Kuchel GA (2007). Geriatric syndromes: clinical, research, and policy implications of a core geriatric concept. J Am Geriatr Soc.

[R82] Cigolle CT, Langa KM, Kabeto MU, Tian Z, Blaum CS (2007). Geriatric conditions and disability: the Health and Retirement Study. Ann Intern Med.

[R83] Sinclair AJ, Conroy SP, Bayer AJ (2008). Impact of diabetes on physical function in older people. Diabetes Care.

[R84] Munshi M, Grande L, Hayes M, Ayres D, Suhl E, Capelson R, Lin S, Milberg W, Weinger K (2006). Cognitive dysfunction is associated with poor diabetes control in older adults. Diabetes Care.

[R85] Ciechanowski PS, Katon WJ, Russo JE (2000). Depression and diabetes: impact of depressive symptoms on adherence, function, and costs. Arch Intern Med.

[R86] Ruis C, Biessels GJ, Gorter KJ, van den Donk M, Kappelle LJ, Rutten GE (2009). Cognition in the early stage of type 2 diabetes. Diabetes Care.

[R87] Yaffe K, Falvey C, Hamilton N, Schwartz AV, Simonsick EM, Satterfield S, Cauley JA, Rosano C, Launer LJ, Strotmeyer ES, Harris TB (2012). Diabetes, glucose control, and 9-year cognitive decline among older adults without dementia. Arch Neurol.

[R88] Punthakee Z, Miller ME, Launer LJ, Williamson JD, Lazar RM, Cukierman-Yaffee T, Seaquist ER, Ismail-Beigi F, Sullivan MD, Lovato LC, Bergenstal RM, Gerstein HC (2012). ACCORD Group of Investigators; ACCORD-MIND Investigators. Poor cognitive function and risk of severe hypoglycemia in type 2 diabetes: post hoc epidemiologic analysis of the ACCORD trial. Diabetes Care.

[R89] Accardi G, Caruso C, Colonna-Romano G, Camarda C, Monastero R, Candore G (2012). Can Alzheimer disease be a form of type 3 diabetes?. Rejuvenation Res.

[R90] Strachan MW, Reynolds RM, Marioni RE, Price JF (2011). Cognitive function, dementia and type 2 diabetes mellitus in the elderly. Nat Rev Endocrinol.

[R91] Kodl CT, Seaquist ER (2008). Cognitive dysfunction and diabetes mellitus. Endocr Rev.

[R92] S Roriz-Filho J, Sá-Roriz TM, Rosset I, Camozzato AL, Santos AC, Chaves ML, Moriguti JC, Roriz-Cruz M (2009). (Pre)diabetes, brain aging, and cognition. Biochim Biophys Acta.

[R93] Lin SJ, Guarente L (2003). Nicotinamide adenine dinucleotide, a metabolic regulator of transcription, longevity and disease. Curr Opin Cell Biol.

[R94] Min SW, Sohn PD, Cho SH, Swanson RA, Gan L (2013). Sirtuins in neurodegenerative diseases: an update on potential mechanisms. Front Aging Neurosci.

[R95] Herskovits AZ, Guarente L (2013). Sirtuin deacetylases in neurodegenerative diseases of aging. Cell Res.

[R96] Song R, Xu W, Chen Y, Li Z, Zeng Y, Fu Y (2011). The expression of Sirtuins 1 and 4 in peripheral blood leukocytes from patients with type 2 diabetes. Eur J Histochem.

[R97] Sun C, Zhang F, Ge X, Yan T, Chen X, Shi X, Zhai Q (2007). SIRT1 improves insulin sensitivity under insulin-resistant conditions by repressing PTP1B. Cell Metab.

[R98] Neumiller JJ, Setter SM (2009). Pharmacologic management of the older patient with type 2 diabetes mellitus. Am J Geriatr Pharmacother.

[R99] Aymanns C, Keller F, Maus S, Hartmann B, Czock D (2010). Review on pharmacokinetics and pharmacodynamics and the aging kidney. Clin J Am Soc Nephrol.

[R100] Shi S, Klotz U (2011). Age-related changes in pharmacokinetics. Curr Drug Metab.

[R101] Nobili A, Pasina L, Tettamanti M, Lucca U, Riva E, Marzona I, Monesi L, Cucchiani R, Bortolotti A, Fortino I, Merlino L, Walter Locatelli G, Giuliani G (2009). Potentially severe drug interactions in elderly outpatients: results of an observational study of an administrative prescription database. J Clin Pharm Ther.

[R102] Chelliah A, Burge MR (2004). Hypoglycaemia in elderly patients with diabetes mellitus: causes and strategies for prevention. Drugs Aging.

[R103] Shorr RI, Ray WA, Daugherty JR, Griffin MR (1997). Incidence and risk factors for serious hypoglycemia in older persons using insulin or sulfonylureas. Arch Intern Med.

[R104] Alagiakrishnan K, Mereu L (2010). Approach to managing hypoglycemia in elderly patients with diabetes. Postgrad Med.

[R105] Amiel SA, Dixon T, Mann R, Jameson K (2008). Hypoglycaemia in Type 2 diabetes. Diabet Med.

[R106] Meneilly GS, Cheung E, Tuokko H (1994). Altered responses to hypoglycemia of healthy elderly people. J Clin Endocrinol Metab.

[R107] Bremer JP, Jauch-Chara K, Hallschmid M, Schmid S, Schultes B (2009). Hypoglycemia unawareness in older compared with middle-aged patients with type 2 diabetes. Diabetes Care.

[R108] Lassmann-Vague V (2005). Hypoglycaemia in elderly diabetic patients. Diabetes Metab.

[R109] Miller ME, Bonds DE, Gerstein HC, Seaquist ER, Bergenstal RM, Calles-Escandon J, Childress RD, Craven TE, Cuddihy RM, Dailey G, Feinglos MN, Ismail-Beigi F, Largay JF (2010). The effects of baseline characteristics, glycaemia treatment approach, and glycated haemoglobin concentration on the risk of severe hypoglycaemia: post hoc epidemiological analysis of the ACCORD study. BMJ.

[R110] Zammitt NN, Frier BM (2005). Hypoglycemia in type 2 diabetes: pathophysiology, frequency, and effects of different treatment modalities. Diabetes Care.

[R111] Whitmer RA, Karter AJ, Yaffe K, Quesenberry CP, Selby JV (2009). Hypoglycemic episodes and risk of dementia in older patients with type 2 diabetes mellitus. JAMA.

[R112] Bonds DE, Miller ME, Bergenstal RM, Buse JB, Byington RP, Cutler JA, Dudl RJ, Ismail-Beigi F, Kimel AR, Hoogwerf B, Horowitz KR, Savage PJ, Seaquist ER (2010). The association between symptomatic, severe hypoglycaemia and mortality in type 2 diabetes: retrospective epidemiological analysis of the ACCORD study. BMJ.

[R113] Barnett AH (2010). Avoiding hypoglycaemia while achieving good glycaemic control in type 2 diabetes through optimal use of oral agent therapy. Curr Med Res Opin.

[R114] Jönsson L, Bolinder B, Lundkvist J (2006). Cost of hypoglycemia in patients with Type 2 diabetes in Sweden. Value Health.

[R115] McEwan P, Evans M, Bergenheim K (2010). A population model evaluating the costs and benefits associated with different oral treatment strategies in people with type 2 diabetes. Diabetes Obes Metab.

[R116] Greco D, Pisciotta M, Gambina F, Maggio F (2010). Severe hypoglycaemia leading to hospital admission in type 2 diabetic patients aged 80 years or older. Exp Clin Endocrinol Diabetes.

[R117] Budnitz DS, Lovegrove MC, Shehab N, Richards CL (2011). Emergency hospitalizations for adverse drug events in older Americans. N Engl J Med.

[R118] Skyler JS, Bergenstal R, Bonow RO, Buse J, Deedwania P, Gale EA, Howard BV, Kirkman MS, Kosiborod M, Reaven P, Sherwin RS (2009). American Diabetes Association; American College of Cardiology Foundation; American Heart Association. Intensive glycemic control and the prevention of cardiovascular events: implications of the ACCORD, ADVANCE, and VA diabetes trials: a position statement of the American Diabetes Association and a scientific statement of the American College of Cardiology Foundation and the American Heart Association. Circulation.

[R119] Miller ME, Williamson JD, Gerstein HC, Byington RP, Cushman WC, Ginsberg HN, Ambrosius WT, Lovato L, Applegate WB (2013). Effects of Randomization to Intensive Glucose Control on Adverse Events, Cardiovascular Disease and Mortality in Older Versus Younger Adults in the ACCORD Trial. Diabetes Care.

[R120] Durso SC (2006). Using clinical guidelines designed for older adults with diabetes mellitus and complex health status. JAMA.

[R121] Pozzilli P, Leslie RD, Chan J, De Fronzo R, Monnier L, Raz I, Del Prato S (2010). The A1C and ABCD of glycaemia management in type 2 diabetes: a physician's personalized approach. Diabetes Metab Res Rev.

[R122] Abdelhafiz AH, Sinclair AJ (2013). Tailor treatment in the older patient with type 2 diabetes. Practitioner.

[R123] Hilmer SN, McLachlan AJ, Le Couteur DG (2007). Clinical pharmacology in the geriatric patient. Fundam Clin Pharmacol.

[R124] Blaum C, Cigolle CT, Boyd C, Wolff JL, Tian Z, Langa KM, Weir DR (2010). Clinical complexity in middle-aged and older adults with diabetes: the Health and Retirement Study. Med Care.

[R125] Laiteerapong N, Iveniuk J, John PM, Laumann EO, Huang ES (2012). Classification of older adults who have diabetes by comorbid conditions, United States, 2005-2006. Prev Chronic Dis.

[R126] Ismail-Beigi F, Moghissi E, Tiktin M, Hirsch IB, Inzucchi SE, Genuth S (2011). Individualizing glycemic targets in type 2 diabetes mellitus: implications of recent clinical trials. Ann Intern Med.

[R127] Lee SJ, Eng C (2011). Goals of glycemic control in frail older patients with diabetes. JAMA.

[R128] U.S. Department of Veterans Affair VA/DOD clinical Practice Guidelines: Management of Diabetes Mellitus in Primary Care (2010). http://www.healthquality.va.gov/Diabetes%20Mellitus.%20Asp.

[R129] Bailey CJ, Turner RC (1996). Metformin. N Engl J Med.

[R130] Rodbard HW, Jellinger PS, Davidson JA, Einhorn D, Garber AJ, Grunberger G, Handelsman Y, Horton ES, Lebovitz H, Levy P, Moghissi ES, Schwartz SS (2009). Statement by an American Association of Clinical Endocrinologists/American College of Endocrinology consensus panel on type 2 diabetes mellitus: an algorithm for glycemic control. Endocr Pract.

[R131] Johnson JA, Majumdar SR, Simpson SH, Toth EL (2002). Decreased mortality associated with the use of metformin compared with sulfonylurea monotherapy in type 2 diabetes. Diabetes Care.

[R132] Rojas LB, Gomes MB (2013). Metformin: an old but still the best treatment for type 2 diabetes. Diabetol Metab Syndr.

[R133] Kirpichnikov D, McFarlane SI, Sowers JR (2002). Metformin: an update. Ann Intern Med.

[R134] Nathan DM (2002). Clinical practice. Initial management of glycemia in type 2 diabetes mellitus. N Engl J Med.

[R135] de Jager J, Kooy A, Lehert P, Wulffelé MG, van der Kolk J, Bets D, Verburg J, Donker AJ, Stehouwer CD (2010). Long term treatment with metformin in patients with type 2 diabetes and risk of vitamin B-12 deficiency: randomised placebo controlled trial. BMJ.

[R136] Wulffelé MG, Kooy A, Lehert P, Bets D, Ogterop JC, Borger van der Burg B, Donker AJ, Stehouwer CD (2003). Effects of short-term treatment with metformin on serum concentrations of homocysteine, folate and vitamin B12 in type 2 diabetes mellitus: a randomized, placebo-controlled trial. J Intern Med.

[R137] Ting RZ, Szeto CC, Chan MH, Ma KK, Chow KM (2006). Risk factors of vitamin B(12) deficiency in patients receiving metformin. Arch Intern Med.

[R138] Lachner C, Steinle NI, Regenold WT (2012). The neuropsychiatry of vitamin B12 deficiency in elderly patients. J Neuropsychiatry Clin Neurosci.

[R139] Rodbard HW, Jellinger PS, Davidson JA, Einhorn D, Garber AJ, Grunberger G, Handelsman Y, Horton ES, Lebovitz H, Levy P, Moghissi ES, Schwartz SS (2009). Statement by an American Association of Clinical Endocrinologists/American College of Endocrinology consensus panel on type 2 diabetes mellitus: an algorithm for glycemic control. Endocr Pract.

[R140] Brown JB, Pedula K, Barzilay J, Herson MK, Latare P (1998). Lactic acidosis rates in type 2 diabetes. Diabetes Care.

[R141] Salpeter SR, Greyber E, Pasternak GA, Salpeter EE (2010). Risk of fatal and nonfatal lactic acidosis with metformin use in type 2 diabetes mellitus. Cochrane Database Syst Rev.

[R142] Solini A, Penno G, Bonora E, Fondelli C, Orsi E, Trevisan R, Vedovato M, Cavalot F, Cignarelli M, Morano S, Ferrannini E, Pugliese G (2013). Renal Insufficiency and Cardiovascular Events Study Group. Age, renal dysfunction, cardiovascular disease, and antihyperglycemic treatment in type 2 diabetes mellitus: findings from the Renal Insufficiency and Cardiovascular Events Italian Multicenter Study. J Am Geriatr Soc.

[R143] Lipska KJ, Bailey CJ, Inzucchi SE (2011). Use of metformin in the setting of mild-to-moderate renal insufficiency. Diabetes Care.

[R144] Frid A, Sterner GN, Londahl M, Wiklander C, Cato A, Vinge E, Andersson A (2010). Novel assay of metformin levels in patients with type 2 diabetes and varying levels of renal function: clinical recommendations. Diabetes Care.

[R145] Yki-Järvinen H (2004). Thiazolidinediones. N Engl J Med.

[R146] Mooradian AD, Chehade J, Thurman JE (2002). The role of thiazolidinediones in the treatment of patients with type 2 diabetes mellitus. Treat Endocrinol.

[R147] Kahn SE, Zinman B, Lachin JM, Haffner SM, Herman WH, Holman RR, Kravitz BG, Yu D, Heise MA, Aftring RP, Viberti G (2008). Diabetes Outcome Progression Trial (ADOPT) Study Group. Rosiglitazone-associated fractures in type 2 diabetes: an Analysis from A Diabetes Outcome Progression Trial (ADOPT). Diabetes Care.

[R148] Meier C, Kraenzlin ME, Bodmer M, Jick SS, Jick H, Meier CR (2008). Use of thiazolidinediones and fracture risk. Arch Intern Med.

[R149] Lewis JD, Ferrara A, Peng T, Hedderson M, Bilker WB, Quesenberry CP, Vaughn DJ, Nessel L, Selby J, Strom BL (2011). Risk of bladder cancer among diabetic patients treated with pioglitazone: interim report of a longitudinal cohort study. Diabetes Care.

[R150] Mamtani R, Haynes K, Bilker WB, Vaughn DJ, Strom BL, Glanz K, Lewis JD (2012). Association between longer therapy with thiazolidinediones and risk of bladder cancer: a cohort study. J Natl Cancer Inst.

[R151] Azoulay L, Yin H, Filion KB, Assayag J, Majdan A, Pollak MN, Suissa S (2012). The use of pioglitazone and the risk of bladder cancer in people with type 2 diabetes: nested case-control study. BMJ.

[R152] Nathan DM, Buse JB, Davidson MB, Ferrannini E, Holman RR, Sherwin R, Zinman B (2009). American Diabetes Association; European Association for Study of Diabetes. Medical management of hyperglycemia in type 2 diabetes: a consensus algorithm for the initiation and adjustment of therapy: a consensus statement of the American Diabetes Association and the European Association for the Study of Diabetes. Diabetes Care.

[R153] Lipscombe LL, Gomes T, Lévesque LE, Hux JE, Juurlink DN, Alter DA (2007). Thiazolidinediones and cardiovascular outcomes in older patients with diabetes. JAMA.

[R154] Home PD, Pocock SJ, Beck-Nielsen H, Curtis PS, Gomis R, Hanefeld M, Jones NP, Komajda M, McMurray JJ (2009). RECORD Study Team. Rosiglitazone evaluated for cardiovascular outcomes in oral agent combination therapy for type 2 diabetes (RECORD): a multicentre, randomised, open-label trial. Lancet.

[R155] Masoudi FA, Inzucchi SE, Wang Y, Havranek EP, Foody JM, Krumholz HM (2005). Thiazolidinediones, metformin, and outcomes in older patients with diabetes and heart failure: an observational study. Circulation.

[R156] Wilcox R, Kupfer S, Erdmann E (2008). PROactive Study investigators. Effects of pioglitazone on major adverse cardiovascular events in high-risk patients with type 2 diabetes: results from PROspective pioglitAzone Clinical Trial In macro Vascular Events (PROactive 10). Am Heart J.

[R157] Dormandy JA, Betteridge DJ, Schernthaner G, Pirags V, Norgren L (2009). PROactive investigators. Impact of peripheral arterial disease in patients with diabetes--results from PROactive (PROactive 11). Atherosclerosis.

[R158] Sato T, Hanyu H, Hirao K, Kanetaka H, Sakurai H, Iwamoto T (2011). Efficacy of PPAR-γ agonist pioglitazone in mild Alzheimer disease. Neurobiol Aging.

[R159] Salvatore T, Giugliano D (1996). Pharmacokinetic-pharmacodynamic relationships of Acarbose. Clin Pharmacokinet.

[R160] Josse RG, Chiasson JL, Ryan EA, Lau DC, Ross SA, Yale JF, Leiter LA, Maheux P, Tessier D, Wolever TM, Gerstein H, Rodger NW, Dornan JM (2003). Acarbose in the treatment of elderly patients with type 2 diabetes. Diabetes Res Clin Pract.

[R161] Yang G, Li C, Gong Y, Li J, Cheng X, Tian H (2013). A prospective, randomized, open-label study comparing the efficacy and safety of preprandial and prandial insulin in combination with acarbose in elderly, insulin-requiring patients with type 2 diabetes mellitus. Diabetes Technol Ther.

[R162] Sturgess NC, Ashford ML, Cook DL, Hales CN (1985). The sulphonylurea receptor may be an ATP-sensitive potassium channel. Lancet.

[R163] Proks P, Reimann F, Green N, Gribble F, Ashcroft F (2002). Sulfonylurea stimulation of insulin secretion. Diabetes.

[R164] Dornhorst A (2001). Insulinotropic meglitinide analogues. Lancet.

[R165] Scott LJ (2012). Repaglinide: a review of its use in type 2 diabetes mellitus. Drugs.

[R166] Cayea D, Boyd C, Durso SC (2007). Individualising therapy for older adults with diabetes mellitus. Drugs Aging.

[R167] Shorr RI, Ray WA, Daugherty JR, Griffin MR (1996). Individual sulfonylureas and serious hypoglycemia in older people. J Am Geriatr Soc.

[R168] Papa G, Fedele V, Rizzo MR, Fioravanti M, Leotta C, Solerte SB, Purrello F, Paolisso G (2006). Safety of type 2 diabetes treatment with repaglinide compared with glibenclamide in elderly people: A randomized, open-label, two-period, cross-over trial. Diabetes Care.

[R169] Germino FW (2011). Noninsulin treatment of type 2 diabetes mellitus in geriatric patients: a review. Clin Ther.

[R170] Riveline JP, Danchin N, Ledru F, Varroud-Vial M, Charpentier G (2003). Sulfonylureas and cardiovascular effects: from experimental data to clinical use. Available data in humans and clinical applications. Diabetes Metab.

[R171] Pantalone KM, Kattan MW, Yu C, Wells BJ, Arrigain S, Jain A, Atreja A, Zimmerman RS (2012). Increase in overall mortality risk in patients with type 2 diabetes receiving glipizide, glyburide or glimepiride monotherapy versus metformin: a retrospective analysis. Diabetes Obes Metab.

[R172] Ahrén B (2003). Gut peptides and type 2 diabetes mellitus treatment. Curr Diab Rep.

[R173] Baggio LL, Drucker DJ (2007). Biology of incretins: GLP-1 and GIP. Gastroenterology.

[R174] Drucker DJ, Nauck MA (2006). The incretin system: glucagon-like peptide-1 receptor agonists and dipeptidyl peptidase-4 inhibitors in type 2 diabetes. Lancet.

[R175] Abbatecola AM, Maggi S, Paolisso G (2008). New approaches to treating type 2 diabetes mellitus in the elderly: role of incretin therapies. Drugs Aging.

[R176] Paolisso G, Monami M, Marfella R, Rizzo MR, Mannucci E (2012). Dipeptidyl peptidase-4 inhibitors in the elderly: more benefits or risks?. Adv Ther.

[R177] Schwartz SL (2010). Treatment of elderly patients with type 2 diabetes mellitus: a systematic review of the benefits and risks of dipeptidyl peptidase-4 inhibitors. Am J Geriatr Pharmacother.

[R178] Pratley RE, Rosenstock J, Pi-Sunyer FX, Banerji MA, Schweizer A, Couturier A, Dejager S (2007). Management of type 2 diabetes in treatment-naive elderly patients: benefits and risks of vildagliptin monotherapy. Diabetes Care.

[R179] Pratley RE, McCall T, Fleck PR, Wilson CA, Mekki Q (2009). Alogliptin use in elderly people: a pooled analysis from phase 2 and 3 studies. J Am Geriatr Soc.

[R180] Blonde L, Dagogo-Jack S, Banerji MA, Pratley RE, Marcellari A, Braceras R, Purkayastha D, Baron M (2009). Comparison of vildagliptin and thiazolidinedione as add-on therapy in patients inadequately controlled with metformin: results of the GALIANT trial--a primary care, type 2 diabetes study. Diabetes Obes Metab.

[R181] Schweizer A, Dejager S, Bosi E (2009). Comparison of vildagliptin and metformin monotherapy in elderly patients with type 2 diabetes: a 24-week, double-blind, randomized trial. Diabetes Obes Metab.

[R182] Halimi S, Raccah D, Schweizer A, Dejager S (2010). Role of vildagliptin in managing type 2 diabetes mellitus in the elderly. Curr Med Res Opin.

[R183] Schweizer A, Dejager S, Foley JE, Shao Q, Kothny W (2011). Clinical experience with vildagliptin in the management of type 2 diabetes in a patient population ≥75 years: a pooled analysis from a database of clinical trials. Diabetes Obes Metab.

[R184] Barzilai N, Guo H, Mahoney EM, Caporossi S, Golm GT, Langdon RB, Williams-Herman D, Kaufman KD, Amatruda JM, Goldstein BJ, Steinberg H (2011). Efficacy and tolerability of sitagliptin monotherapy in elderly patients with type 2 diabetes: a randomized, double-blind, placebo-controlled trial. Curr Med Res Opin.

[R185] Karyekar CS, Ravichandran S, Allen E, Fleming D, Frederich R (2013). Tolerability and efficacy of glycemic control with saxagliptin in older patients (aged ≥ 65 years) with inadequately controlled type 2 diabetes mellitus. Clin Interv Aging.

[R186] Barnett AH, Huisman H, Jones R, von Eynatten M, Patel S, Woerle HJ (2013). Linagliptin for patients aged 70 years or older with type 2 diabetes inadequately controlled with common antidiabetes treatments: a randomised, double-blind, placebo-controlled trial. Lancet.

[R187] Deacon CF, Holst JJ (2010). Linagliptin, a xanthine-based dipeptidyl peptidase-4 inhibitor with an unusual profile for the treatment of type 2 diabetes. Expert Opin Investig Drugs.

[R188] Lukashevich V, Schweizer A, Shao Q, Groop PH, Kothny W (2011). Safety and efficacy of vildagliptin versus placebo in patients with type 2 diabetes and moderate or severe renal impairment: a prospective 24-week randomized placebo-controlled trial. Diabetes Obes Metab.

[R189] Russo E, Penno G, Del Prato S (2013). Managing diabetic patients with moderate or severe renal impairment using DPP-4 inhibitors: focus on vildagliptin. Diabetes Metab Syndr Obes.

[R190] Eligar VS, Bain SC (2013). A review of sitagliptin with special emphasis on its use in moderate to severe renal impairment. Drug Des Devel Ther.

[R191] Nowicki M, Rychlik I, Haller H, Warren ML, Suchower L, Gause-Nilsson I (2011). D1680C00007 Investigators. Saxagliptin improves glycaemic control and is well tolerated in patients with type 2 diabetes mellitus and renal impairment. Diabetes Obes Metab.

[R192] Friedrich C, Emser A, Woerle HJ, Graefe-Mody U (2013). Renal impairment has no clinically relevant effect on the long-term exposure of linagliptin in patients with type 2 diabetes. Am J Ther.

[R193] Strain WD, Lukashevich V, Kothny W, Hoellinger MJ, Paldánius PM (2013). Individualised treatment targets for elderly patients with type 2 diabetes using vildagliptin add-on or lone therapy (INTERVAL): a 24 week, randomised, double-blind, placebo-controlled study. Lancet.

[R194] Ligueros-Saylan M, Foley JE, Schweizer A, Couturier A, Kothny W (2010). An assessment of adverse effects of vildagliptin versus comparators on the liver, the pancreas, the immune system, the skin and in patients with impaired renal function from a large pooled database of Phase II and III clinical trials. Diabetes Obes Metab.

[R195] Del Prato S, Barnett AH, Huisman H, Neubacher D, Woerle HJ, Dugi KA (2011). Effect of linagliptin monotherapy on glycaemic control and markers of β-cell function in patients with inadequately controlled type 2 diabetes: a randomized controlled trial. Diabetes Obes Metab.

[R196] Gomis R, Espadero RM, Jones R, Woerle HJ, Dugi KA (2011). Efficacy and safety of initial combination therapy with linagliptin and pioglitazone in patients with inadequately controlled type 2 diabetes: a randomized, double-blind, placebo-controlled study. Diabetes Obes Metab.

[R197] Taskinen MR, Rosenstock J, Tamminen I, Kubiak R, Patel S, Dugi KA, Woerle HJ (2011). Safety and efficacy of linagliptin as add-on therapy to metformin in patients with type 2 diabetes: a randomized, double-blind, placebo-controlled study. Diabetes Obes Metab.

[R198] Owens DR, Swallow R, Dugi KA, Woerle HJ (2011). Efficacy and safety of linagliptin in persons with type 2 diabetes inadequately controlled by a combination of metformin and sulphonylurea: a 24-week randomized study. Diabet Med.

[R199] Gallwitz B, Rosenstock J, Rauch T, Bhattacharya S, Patel S, von Eynatten M, Dugi KA, Woerle HJ (2012). 2-year efficacy and safety of linagliptin compared with glimepiride in patients with type 2 diabetes inadequately controlled on metformin: a randomised, double-blind, non-inferiority trial. Lancet.

[R200] Scheen AJ (2013). Cardiovascular effects of dipeptidyl peptidase-4 inhibitors: from risk factors to clinical outcomes. Postgrad Med.

[R201] D'Amico M, Di Filippo C, Marfella R, Abbatecola AM, Ferraraccio F, Rossi F, Paolisso G (2010). Long-term inhibition of dipeptidyl peptidase-4 in Alzheimer's prone mice. Exp Gerontol.

[R202] Darsalia V, Ortsäter H, Olverling A, Darlöf E, Wolbert P, Nyström T, Klein T, Sjöholm Å, Patrone C (2013). The DPP-4 inhibitor linagliptin counteracts stroke in the normal and diabetic mouse brain: a comparison with glimepiride. Diabetes.

[R203] Pintana H, Apaijai N, Chattipakorn N, Chattipakorn SC (2013). DPP-4 inhibitors improve cognition and brain mitochondrial function of insulin-resistant rats. J Endocrinol.

[R204] Pipatpiboon N, Pintana H, Pratchayasakul W, Chattipakorn N, Chattipakorn SC (2013). DPP4-inhibitor improves neuronal insulin receptor function, brain mitochondrial function and cognitive function in rats with insulin resistance induced by high-fat diet consumption. Eur J Neurosci.

[R205] Scirica BM, Bhatt DL, Braunwald E, Steg PG, Davidson J, Hirshberg B, Ohman P, Frederich R, Wiviott SD, Hoffman EB, Cavender MA, Udell JA, Desai NR, Mosenzon O, McGuire DK, Ray KK, Leiter LA, Raz I (2013). SAVOR-TIMI 53 Steering Committee and Investigators. Saxagliptin and cardiovascular outcomes in patients with type 2 diabetes mellitus. N Engl J Med.

[R206] White WB, Cannon CP, Heller SR, Nissen SE, Bergenstal RM, Bakris GL, Perez AT, Fleck PR, Mehta CR, Kupfer S, Wilson C, Cushman WC, Zannad F (2013). EXAMINE Investigators. Alogliptin after acute coronary syndrome in patients with type 2 diabetes. N Engl J Med.

[R207] Samson SL, Garber A (2013). GLP-1R agonist therapy for diabetes: benefits and potential risks. Curr Opin Endocrinol Diabetes Obes.

[R208] Abbatecola AM, Olivieri F, Corsonello A, Strollo F, Fumagalli A, Lattanzio F (2012). Frailty and safety: the example of diabetes. Drug Saf.

[R209] Linnebjerg H, Kothare PA, Seger M, Wolka AM, Mitchell MI (2011). Exenatide - pharmacokinetics, pharmacodynamics, safety and tolerability in patients ≥ 75 years of age with Type 2 diabetes. Int J Clin Pharmacol Ther.

[R210] Bode BW, Brett J, Falahati A, Pratley RE (2011). Comparison of the efficacy and tolerability profile of liraglutide, a once-daily human GLP-1 analog, in patients with type 2 diabetes ≥65 and &lt;65 years of age: a pooled analysis from phase III studies. Am J Geriatr Pharmacother.

[R211] Scott LJ (2013). Lixisenatide: a review of its use in patients with type 2 diabetes mellitus. BioDrugs.

[R212] Elkinson S, Keating GM (2013). Lixisenatide: first global approval. Drugs.

[R213] Duarte AI, Candeias E, Correia SC, Santos RX, Carvalho C, Cardoso S, Plácido A, Santos MS, Oliveira CR, Moreira PI (2013). Crosstalk between diabetes and brain: glucagon-like peptide-1 mimetics as a promising therapy against neurodegeneration. Biochim Biophys Acta.

[R214] Hunter K, Hölscher C (2012). Drugs developed to treat diabetes, liraglutide and lixisenatide, cross the blood brain barrier and enhance neurogenesis. BMC Neurosci.

[R215] DeFronzo RA, Davidson JA, Del Prato S (2012). The role of the kidneys in glucose homeostasis: a new path towards normalizing glycaemia. Diabetes Obes Metab.

[R216] Ferrannini E, Solini A (2012). SGLT2 inhibition in diabetes mellitus: rationale and clinical prospects. Nat Rev Endocrinol.

[R217] Raskin P (2013). Sodium-glucose cotransporter inhibition: therapeutic potential for the treatment of type 2 diabetes mellitus. Diabetes Metab Res Rev.

[R218] Bode B, Stenlöf K, Sullivan D, Fung A, Usiskin K (2013). Efficacy and safety of canagliflozin treatment in older subjects with type 2 diabetes mellitus: a randomized trial. Hosp Pract (1995).

[R219] Nyirjesy P, Zhao Y, Ways K, Usiskin K (2012). Evaluation of vulvovaginal symptoms and Candida colonization in women with type 2 diabetes mellitus treated with canagliflozin, a sodium glucose co-transporter 2 inhibitor. Curr Med Res Opin.

[R220] Riser Taylor S, Harris KB (2013). The clinical efficacy and safety of sodium glucose cotransporter-2 inhibitors in adults with type 2 diabetes mellitus. Pharmacotherapy.

[R221] Lamos EM, Younk LM, Davis SN (2013). Canagliflozin, an inhibitor of sodium-glucose cotransporter 2, for the treatment of type 2 diabetes mellitus. Expert Opin Drug Metab Toxicol.

[R222] Yale JF, Bakris G, Cariou B, Yue D, David-Neto E, Xi L, Figueroa K, Wajs E, Usiskin K, Meininger G (2013). Efficacy and safety of canagliflozin in subjects with type 2 diabetes and chronic kidney disease. Diabetes Obes Metab.

[R223] Gunasekaran U, Gannon M (2011). Type 2 diabetes and the aging pancreatic beta cell. Aging (Albany NY).

[R224] Tanwani LK (2011). Insulin therapy in the elderly patient with diabetes. Am J Geriatr Pharmacother.

[R225] Reza M, Taylor CD, Towse K, Ward JD, Hendra TJ (2002). Insulin improves well-being for selected elderly type 2 diabetic subjects. Diabetes Res Clin Pract.

[R226] Rosenstock J, Schwartz SL, Clark CM, Park GD, Donley DW, Edwards MB (2001). Basal insulin therapy in type 2 diabetes: 28-week comparison of insulin glargine (HOE 901) and NPH insulin. Diabetes Care.

[R227] Hermansen K, Davies M, Derezinski T, Martinez Ravn G, Clauson P, Home P (2006). A 26-week, randomized, parallel, treat-to-target trial comparing insulin detemir with NPH insulin as add-on therapy to oral glucose-lowering drugs in insulin-naive people with type 2 diabetes. Diabetes Care.

[R228] Philis-Tsimikas A, Charpentier G, Clauson P, Ravn GM, Roberts VL, Thorsteinsson B (2006). Comparison of once-daily insulin detemir with NPH insulin added to a regimen of oral antidiabetic drugs in poorly controlled type 2 diabetes. Clin Ther.

[R229] Lee P, Chang A, Blaum C, Vlajnic A, Gao L, Halter J (2012). Comparison of safety and efficacy of insulin glargine and neutral protamine hagedorn insulin in older adults with type 2 diabetes mellitus: results from a pooled analysis. J Am Geriatr Soc.

[R230] Janka HU (2007). Combination of oral antidiabetic agents with basal insulin versus premixed insulin alone in randomized elderly patients with type 2 diabetes mellitus. J Am Geriatr Soc.

[R231] Drab SR, Philis-Tsimikas A (2013). A New Option for Glycemic Control: Insulin Degludec, a New-Generation Basal Insulin with an Ultralong Duration of Action. Pharmacotherapy.

[R232] Sorli C, Warren M, Oyer D, Mersebach H, Johansen T, Gough SC (2013). Elderly Patients with Diabetes Experience a Lower Rate of Nocturnal Hypoglycaemia with Insulin Degludec than with Insulin Glargine: A Meta-Analysis of Phase IIIa Trials. Drugs Aging.

[R233] Ligthelm RJ, Gylvin T, DeLuzio T, Raskin P (2011). A comparison of twice-daily biphasic insulin aspart 70/30 and once-daily insulin glargine in persons with type 2 diabetes mellitus inadequately controlled on basal insulin and oral therapy: a randomized, open-label study. Endocr Pract.

[R234] Mannucci E, Cremasco F, Romoli E, Rossi A (2011). The use of insulin in elderly patients with type 2 diabetes mellitus. Expert Opin Pharmacother.

[R235] Kitada M, Koya D (2013). SIRT1 in Type 2 Diabetes: Mechanisms and Therapeutic Potential. Diabetes Metab J.

[R236] Yoshizaki T, Milne JC, Imamura T, Schenk S, Sonoda N, Babendure JL, Lu JC, Smith JJ, Jirousek MR, Olefsky JM (2009). SIRT1 exerts anti-inflammatory effects and improves insulin sensitivity in adipocytes. Mol Cell Biol.

[R237] Yoshizaki T, Schenk S, Imamura T, Babendure JL, Sonoda N, Bae EJ, Oh DY, Lu M, Milne JC, Westphal C, Bandyopadhyay G, Olefsky JM (2010). SIRT1 inhibits inflammatory pathways in macrophages and modulates insulin sensitivity. Am J Physiol Endocrinol Metab.

[R238] Libri V, Brown AP, Gambarota G, Haddad J, Shields GS, Dawes H, Pinato DJ, Hoffman E, Elliot PJ, Vlasuk GP, Jacobson E, Wilkins MR, Matthews PM (2012). A pilot randomized, placebo controlled, double blind phase I trial of the novel SIRT1 activator SRT2104 in elderly volunteers. PLoS One.

[R239] Sue Kirkman M, Briscoe VJ, Clark N, Florez H, Haas LB, Halter JB, Huang ES, Korytkowski MT, Munshi MN, Odegard PS, Pratley RE, Swift CS (2012). Consensus Development Conference on Diabetes and Older Adults. Diabetes in older adults: a consensus report. J Am Geriatr Soc.

